
JOURNAL INFORMATION
==============================
NLM Title Abbreviation: ScientificWorldJournal
Journal ID: ScientificWorldJournal
NLM Journal ID: 101131163
Journal ID: TSWJ

The Scientific World Journal

ISSN: 2356-6140
EISSN: 1537-744X
Publisher: Wiley

ARTICLE INFORMATION
==============================
PMCID: PMC6084380
PMID: 30147567
DOI: 10.1100/tsw.2001.192
Article ID: 862037
Article version: 1
Subjects: Short Report

Transferrin Ensures Survival of Ovarian Carcinoma Cells Subjected to Deferoxamine, TNFA, FASL, Trail or MYC-Activation

Rosenberger Georg 1
Grusch Michael 1
Fuhrmann Gerhard 1
Leuhuber Katharina 1
Polgar Doris 1
Krupitza Georg 1 2 *
Krupitza Georg 1
1 Institute of Clinical Pathology, University of Vienna, A-1090 Vienna, Austria
2 Institute of Zoology, University of Vienna, A-1090 Vienna, Austria
*Georg Krupitza: g.krupitza@akh-wien.ac.at
Publication date: 2001
Electronic publication date: 2001 May 12
Volume: 1
First page: 93
Last page: 93
Copyright: Copyright © 2001 Georg Rosenberger et al.
Copyright year: 2001
License: This is an open access article distributed under the Creative Commons Attribution License, which permits unrestricted use, distribution, and reproduction in any medium, provided the original work is properly cited.
License URL: https://creativecommons.org/licenses/by/3.0/

Funding: Austrian National Bank Fond 7220

==============================
Miami *Nature Biotechnology* Short Reports
*TheScientificWorld* (2001) 1 (S3), 53SR
ISSN 1532-2246; DOI 10.1100/tsw.2001.192

### TRANSFERRIN ENSURES SURVIVAL OF OVARIAN CARCINOMA CELLS SUBJECTED TO DEFEROXAMINE, TNFA, FASL, TRAIL OR *MYC*-ACTIVATION

Sandra Fassl [1], Georg Rosenberger[1], Michael Grusch[1], Gerhard Fuhrmann[1], Katharina Leuhuber[1,2], Doris Polgar[1], and Georg Krupitza[1*]

[1]Institute of Clinical Pathology; [2]Institute of Zoology; University of Vienna, A-1090 Vienna, Austria
*g.krupitza@akh-wien.ac.at

**INTRODUCTION.** Human ovarian adenocarcinoma N.1 cells apoptose upon *Myc*-activation when serum factors are limited (1). The downstream mechanism that is triggered by Myc is unknown, however. We discovered that *Myc*-activation and treatment with TNFa, FasL and TRAIL induces H-ferritin expression. H-ferritin is an intracellular iron chelator. Since also the pharmacological iron chelator deferoxamine induces apoptosis in N.1 cells, we investigated the regulation and the role of H-ferritin in response to *Myc*-activation and TNFa treatment under serum free, pro-apoptotic conditions, and identified transferrin as a survival factor in N.1 human ovarian carcinoma cells.

**METHODS.** Western blotting, northern blotting and DNA fragmentation and apoptosis assays were performed according to standard procedures and transfection and retroviral transduction according to standard protocols.

**RESULTS.** When N.1 cells are treated with TRAIL, FasL or TNFa under serum deprived cell culture conditions, which is a prerequisite to induce apoptosis, H-ferritin becomes upregulated (Fig. 1a). Under non-apoptotic conditions, i.e. when cells are maintained under 10% fetal calf serum, the treatment with these inducers prevents cell death (2) and also H-ferritin levels are not induced (Fig. 1b). Activation of an ectopic Myc:ER construct also induces H-ferritin expression when serum is withdrawn, not however in presence of serum. Therefore we assumed that intracellular iron sequestration might be a trigger mechanism of Myc-induced apoptosis. Hence, transferrin was substituted for serum and this, in fact, rescues from apoptosis and does not allow for H-ferritin induction (Fig. 1c). Substitution of transferrin for serum also aborts cell death elicited by TNFa, FasL and TRAIL (Fig. 2), and moreover, prevents downregulation of Akt during TNFa-treatment.

**Fig 1**

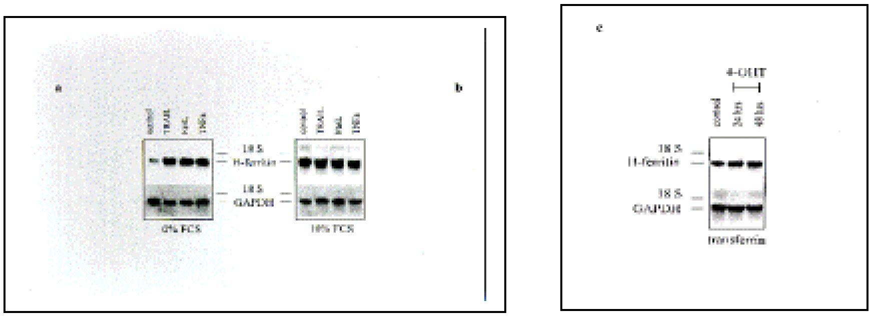

**Fig 2**

Cells were grown to 20% confluence, then serum was withdrawn. 24 hours after serum deprivation transferrin (TF; 10µg/ml) was added and after 5 hours TNFα (50ng/ml), inducing Fas antibody (FasAb; 80ng/ml) or TRAIL (75ng/ml; TRAIL-enhancer: 1µg/ml) were added for 72 hours. Cells floating in the supernatant were pooled with cells, which were trypsinized from the culture dish. Apoptosis rates were determined by HO/PI staining.

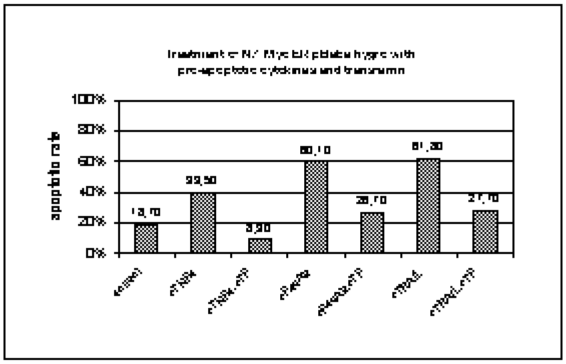

**CONCLUSION.** These data suggest that *Myc*-activation, FasL, TNFa and TRAIL disturb cellular iron homeostasis and that this is an apoptotic trigger. TNFa suppresses constitutive levels of Akt and phosphorylated Akt, which are restored in presence of transferrin. Downregulation of Akt might generate an apoptosis-prone state in N.1 cells by inhibiting a major survival pathway.

It was shown recently that H-ferritin is a target of c-Myc which downregulates its expression under high serum conditions and thereby induces proliferation (3). It can be speculated whether the distinct regulation of H-ferritin by Myc is a switch for the dual pathway that either promotes Myc-triggered growth or apoptosis, depending on survival factor availability.

**ACKNOWLEDGEMENT.** This work was supported by the Austrian National Bank Fond No.: 7220, the Herzfelder´sche family foundation, and the Anton Dreher memory foundation.

**REFERENCES.**

1. Fuhrmann, G., Rosenberger, G., Grusch, M., Klein, N., Hofmann, J., and Krupitza, G. (1999) Mutat. Res. 437, 205-217
2. Simonitsch, I. and Krupitza, G. (1998) Br. J. Cancer 78, 862-870
3. Wu, K., Polack, A., and Dalla-Favera, R. (1999) Science 283, 676-679
